# Multivariate Modeling of Student Performance on NBME Subject Exams

**DOI:** 10.7759/cureus.40809

**Published:** 2023-06-22

**Authors:** Seth M Alexander, Christina L Shenvi, Kimberley R Nichols, Georgette Dent, Kelly L Smith

**Affiliations:** 1 Medicine, University of North Carolina at Chapel Hill School of Medicine, Chapel Hill, USA; 2 Education, Harvard Graduate School of Education, Cambridge, USA; 3 Emergency Medicine, University of North Carolina at Chapel Hill School of Medicine, Chapel Hill, USA; 4 Anesthesiology, University of North Carolina at Chapel Hill School of Medicine, Chapel Hill, USA; 5 Pathology and Laboratory Medicine, University of North Carolina at Chapel Hill School of Medicine, Chapel Hill, USA; 6 Family Medicine, University of North Carolina at Chapel Hill School of Medicine, Chapel Hill, USA

**Keywords:** nbme, undergraduate medical education, statistical modeling, usmle, medical education

## Abstract

Aim

This study sought to determine whether it was possible to develop statistical models which could be used to accurately correlate student performance on clinical subject exams based on their National Board of Medical Examiner (NBME) self-assessment performance and other variables, described below, as such tools are not currently available.

Methods

Students at a large public medical school were provided fee vouchers for NBME self-assessments before clinical subject exams. Multivariate regression models were then developed based on how self-assessment performance correlated to student success on the subsequent subject exam (Medicine, Surgery, Family Medicine, Obstetrics-Gynecology, Pediatrics, and Psychiatry) while controlling for the proximity of the self-assessment to the exam, USMLE Step 1 score, and the academic quarter.

Results

The variables analyzed satisfied the requirements of linear regression. The correlation strength of individual variables and overall models varied by discipline and outcome (equated percent correct or percentile, Model R^2^ Range: 0.1799-0.4915). All models showed statistical significance on the Omnibus F-test (p<0.001).

Conclusion

The correlation coefficients demonstrate that these models have weak to moderate predictive value, dependent on the clinical subject, in predicting student performance; however, this varies widely based on the subject exam in question. The next step is to utilize these models to identify struggling students to determine if their use reduces failure rates and to further improve model accuracy by controlling for additional variables.

## Introduction

The National Board of Medical Examiners’ (NBME) subject exams remain frequent tools in determining clerkship grades and differentiating among students within an institution [1,2]. Concerns have been raised that the transition to Pass/Fail grading of the United States Medical Licensing Exams (USMLE) Step 1 will emphasize clerkship grades and the USMLE Step 2 exam as key differentiators in residency selection [3-6].

With the continued use of graded assessments in medical education, educators must consider how to identify and then support students who encounter academic difficulties. The use of methods such as tutoring, improved access to neuropsychological evaluation, and USMLE postponements have all been cited as ways to help students when they are falling behind their peer group [7,8]. However, there is no consensus as to how to identify which students may be struggling and benefit from these services. With the emphasis of subject exam scores on course grading, many institutions (including our own) use self-assessment cutoffs that have not been previously validated to identify struggling students. To better predict student success on other forms of assessments (i.e. licensing exams), some groups have turned to complex data modeling, including progress testing, Medical College Admission Test (MCAT) scores, and more, as evidence-driven means of predicting student scores and proactively engaging with struggling students [9-11]. This approach and the use of predictive analytics are complicated by the fact the NBME states that their self-assessments are not predictive tools and do not provide sufficient guidance on score interpretation [12].

As such, this study sought to understand if multivariate statistical modeling (linear regression in this case) can produce a statistical model that predicts student performance on individual NBME subject exams based on available self-assessments. This work was previously presented as a poster at the Association of American Medical Colleges, Southern Group on Educational Affairs 2023 Annual Meeting on March 24, 2023.

## Materials and methods

The University of North Carolina School of Medicine (UNC SOM) transitioned in the academic year 2020-2021 to provide students with vouchers for an NBME Clinical Mastery Series Self-Assessments (herein, Self-Assessments) for each of the six subject exams taken during their clerkship year: Internal Medicine, Surgery, Pediatrics, Obstetrics-Gynecology, Psychiatry, and Family Medicine. 

As part of their subject exam preparation, students are encouraged to take at least one self-assessment during each clerkship. Faculty specify, based on the specific clerkship, when these should be completed (ranging from 2-4 weeks prior to the subject exam); however, students ultimately take the assessment at their convenience. When students used the vouchers funded by the school to take the practice exam, the scores were transmitted to the students and faculty in the SOM’s Office of Academic Excellence (OAE). The OAE is an academic support office that both remediates students experiencing academic difficulties, and proactively gives students information and resources to succeed in medical school. In this case, OAE faculty review students’ self-assessment scores to determine if academic interventions, such as coaching, working with a student tutor, or delaying the subject exams to allow more time to study are needed.

The self-assessments and subsequent subject exam performance data were analyzed using STATA BE Version 17.0 (StataCorp LLC, College Station, TX) to create a multivariable linear regression model. Student success on NBME subject exams (determined by the equated percent of questions correct or percentile on the subject exam) was the predicted variable of these models. Variables considered in the model include self-assessment score, how many days prior to the subject exam the self-assessment was actually taken, the quarter in which the self-assessment and subject exams were taken, and prior performance on the USMLE Step 1. Each model was evaluated to confirm that it did not grossly violate the assumptions of multivariate regression, including normality, linearity, homoscedasticity, and lack of collinearity.

Data were collected and analyzed using multivariate linear regression modeling. Different models were created for each of the six NBME subject exams to determine which model best accounts for the variability in student performance based on the four variables above and using the omnibus F-test as a marker of statistical significance for the model. 

This study was reviewed by the Institutional Review Board of the University of North Carolina at Chapel Hill, which determined it to be exempt from human subjects research regulations and waived the need for informed consent (IRB Study 22-0524).

## Results

The regression model was developed based on subject exam scores and the four variables: self-assessment score, how many days before the subject exam the self-assessment was taken, the quarter in which the exams were taken, and score on the USMLE Step 1. The variability accounted for in each model, as measured by the regression coefficient, varied widely across disciplines, and whether percentile (R^2^ Range 0.1799-0.4568) or equated percent correct (R^2^ Range 0.2142-0.4915) was the predicted outcome. An example of a taxonomy table for the Internal Medicine subject exam is seen in Table 1. The complete models were used to generate equations that can subsequently help predict student scores on the subject exams (Equation 1, below).

**Table 1:** Taxonomy of regression models describing the relationship of medicine subject exam percent of questions correct to multiple variables.

Predictor	Model A	Model B	Model C	Model D	Model E	Model F	Model G
Intercept	39.47*** (9.79)	78.04*** (88.59)	71.46*** (43.33)	24.15** (3.34)	38.94*** (9.29)	36.84*** (8.98)	7.50 (1.13)
Medicine Self-Assessment Score	1.81*** (9.53)				1.82*** (9.47)	1.72*** (9.12)	0.94*** (4.77)
Dates Prior to Subject Exam		-0.02 (-0.76)			0.01 (0.48)	0.00 (0.13)	0.11* (2.26)
Quarter			2.12*** (3.98)			1.53** (3.48)	1.58*** (4.01)
USMLE Step 1 Score				0.23*** (7.63)			0.19 (6.50)
R^2^	0.3522	0.4456	0.0866	0.2965	0.3531	0.3972	0.4915
F	90.79	0.58	15.83	58.17	45.30	36.25	32.63
(df)	(1, 167)	(1, 167)	(1, 167)	(1, 138)	(2, 166)	(3, 165)	(4, 135)
p	<0.0001	0.4456	0.0001	<0.0001	<0.0001	<0.0001	<0.0001
RMSE	6.5941	8.1785	7.8301	5.9547	6.6093	6.3992	5.1184
Each cell lists estimated regression coefficients and (t-statistic). * p < 0.05, ** p < 0.01, *** p < 0.001							

Equation 1

Equated % Correct = 0.94 x SA + 0.11 x T + 1.58 x Q + 0.19 x S1 +7.50

This equation represents the regression model to predict a student’s equated percent correct on the Internal Medicine subject exam based on their self-assessment score (SA), days before the subject exam (T), the quarter (Q), USMLE Step 1 score (S1) (R^2^ = 0.4915, F (4, 135) = 32.63, p<0.001). 

**Figure 1:**
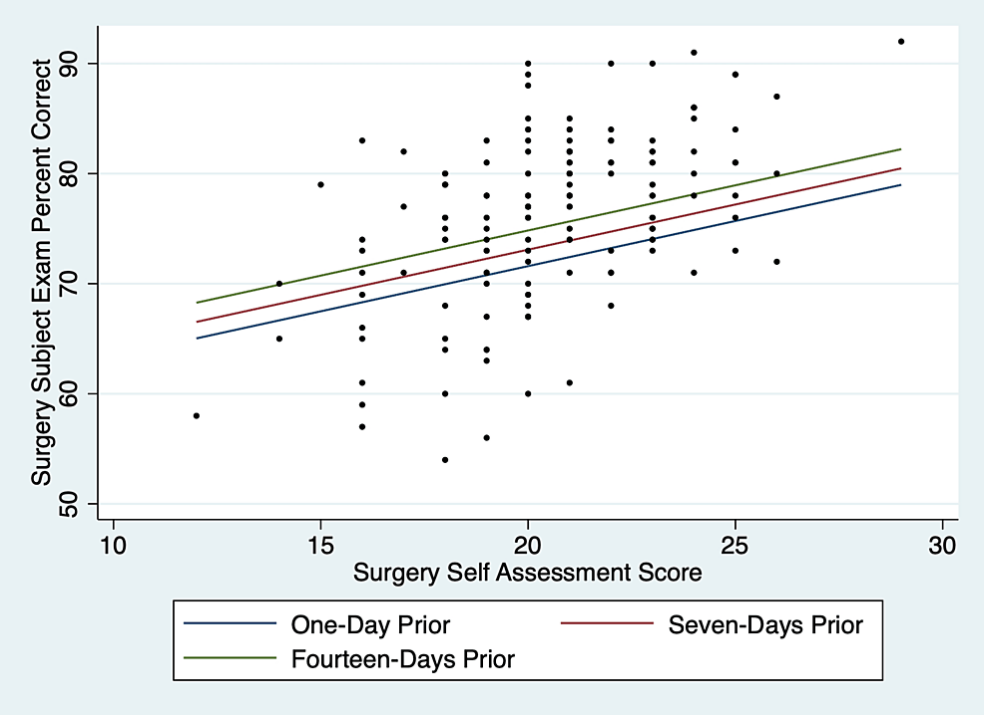
Scatterplot and multiple regression lines predicting surgery subject exam equated percent of questions correct based on a surgery self-assessment taken in the first quarter of the academic year by students who scored the median value on the USMLE Step 1 Exam (237), taking the number of days between self-assessment and the subject exam into account. This graph is duplicated in Figure 5 of the supplemental digital material.

When considering univariate regression models based on the variables across disciplines, the self-assessment score (R^2 ^Range 0.1072-0.3522) and USMLE Step 1 score (R^2^ Range 0.1682-0.2965) were most predictive of the final subject exam scores. These two variables were consistently statistically significant (p < 0.05). Further, additional variables in the linear models were intermittently significant. The quarter was disregarded as a variable from models where subject exam percentile was the outcome because the NBME norms percentiles are based on academic quarters. An example of the multivariate graphs produced by the models can be seen in Figure 1.

## Discussion

While both percentile and equated percent correct outcomes were statistically significant using multivariate regression, the variables in equated percent correct models accounted for more variability than in percentile models, as seen by the R^2^ ranges (R^2^ 0.1072-0.3522). While many programs, including UNC SOM, may use percentile cutoffs to determine academic advancement, these scores are normed by the NBME annually [13]. For this reason, the equated percent correct models are favored because they are statistically more robust and remain easily interpretable despite changes in national norms. Although it is not perfect, this study showed that regression modeling of self-assessment scores can be viewed as a helpful tool to help predict subject exam scores in a way that has not previously been described.

The prediction slightly improves when the Step 1 score is incorporated into the linear model. Given pre-existing data on standardized test scores potentially predicting future standardized test performance [11], the predictive nature of Step 1 was not an entirely surprising finding. However, these authors acknowledge concerns that the USMLE Step 1 is a potentially biased tool [14] and that some students who scored lower on USMLE Step 1 are often coached to achieve a passing score. Step 1 scores are no longer a viable metric for most students given the transition to pass-fail grading [3]; however as shown, the inclusion of additional variables may improve the model further.

Following the development of statistical models to predict student success, the logical conclusion is to apply these data to students actively preparing for the subject exams to help determine which students faculty can approach proactively with academic support structures like tutoring or coaching. In doing so, these statistical models must demonstrate that their predictive value exceeds that of more conventional, subjective means. Following verification, these data and models can be used to drive decisions regarding individual students based on their predicted scores and to drive institutional decisions regarding policies of academic advancement and standardized testing delays for students considered at high risk of subject exam failure. 

It is also important to note that the interpretation of these models has significant limitations. In addition to the previously mentioned variables that are difficult to account for in a simple linear regression model, these models are based on a single-center, retrospective study. Additional data collection from other institutions may help improve the validity of the models by including a larger cohort of students. One of the most predictive variables, performance on the USMLE Step 1 Exam, is also no longer available as a metric given the change in scoring to a pass-fail system in 2022 [15] and therefore cannot be used in applying some of the specific models described herein. There are also other variables, such as the availability of study time in each clerkship, that may be important to student performance but are not easily quantifiable and may not meet the requirements of linear regression (i.e. homoscedasticity). Medical educators will need to think critically about other metrics to help identify students who may need support and/or who could be at risk of future academic difficulty.

Linear regression is also limited in the scope of variables that can be included in the model. Variables that could impact the reliability of the models, such as self-assessment form version and more abstract life circumstances, are particularly difficult to account for in a statistical model. More robust modeling methods such as neural network models and other machine learning models could help further improve the accuracy of predicted outcomes.

## Conclusions

Given the high stakes nature of subject exams, programs should adopt a mechanism by which they can leverage available data to predict which students are at highest risk of failure. This study showed that multivariate regression, while imperfect, can serve as a potential tool to predict student performance. At the same time, it is important to recognize and consider the limitations of these models in the context of each individual student's academic and career advising. Using statistical analysis, institutions may be able to create simple yet powerful predictive tools to help guide conversations around student support and guide national conversations regarding grading, student advancement, and other policy initiatives.

## References

[1] Hernandez CA, Daroowalla F, LaRochelle JS (2021). Determining grades in the internal medicine clerkship: results of a national survey of clerkship directors. Acad Med.

[2] Mattson C, Park YS (2020). Toward thoughtful use of shelf exam scores in clerkship assessment systems. Acad Med.

[3] Cottrell S, Ferrari N (2019). More about USMLE Step 1 scoring. Acad Med.

[4] Ryan MS, Colbert-Getz JM, Glenn SN, Browning JD, Anand RJ (2017). Does the NBME Surgery Shelf exam constitute a "double jeopardy" of USMLE Step 1 performance?. Am J Surg.

[5] Volk LE, Labiner HE, Toussaint A, Maloney Patel N, Nieman DR (2022). Perils of pass-fail: clerkship Shelf Scores are not good surrogates for Step 1. Global Surg Educ.

[6] Quinn KM, Campbell L, Mukherjee R, Abbott AM, Streck CJ (2022). Step 1 is pass/fail, now what? Can clinical clerkship grades be used as a reliable metric to screen general surgery residency applicants?. J Surg Res.

[7] Swan Sein A, Daniel M, Fleming A (2020). Identifying and supporting students to prevent USMLE Step 1 failures when testing follows clerkships: insights from 9 schools. Acad Med.

[8] Alexander SM, Dallaghan GL, Birch M, Smith KL, Howard N, Shenvi CL (2022). What makes a near-peer learning and tutoring program effective in undergraduate medical education: a qualitative analysis. Med Sci Educ.

[9] Wang L, Laird-Fick HS, Parker CJ, Solomon D (2021). Using Markov chain model to evaluate medical students' trajectory on progress tests and predict USMLE step 1 scores---a retrospective cohort study in one medical school. BMC Med Educ.

[10] Schuwirth LW, van der Vleuten CP (2012). The use of progress testing. Perspect Med Educ.

[11] Hanson JT, Busche K, Elks ML (2022). The Validity of MCAT Scores in predicting students' performance and progress in medical school: results from a multisite study. Acad Med.

[12] (2006). National Board of Medical Examiners: NBME Self-Assessment Services Voucher Program Guide. https://www.nbme.org/sites/default/files/2022-12/NBME_NSAS_Voucher_Program_Guide.pdf.

[13] (2023). National Board of Medical Examiners: Subject Examinations. https://www.nbme.org/assessment-products/assess-learn/subject-exams.

[14] Edmond MB, Deschenes JL, Eckler M, Wenzel RP (2001). Racial bias in using USMLE step 1 scores to grant internal medicine residency interviews. Acad Med.

[15] (2022). United States Medical Licensing Exam: USMLE Step 1 Transition to Pass/Fail Only Score Reporting. https://www.usmle.org/usmle-step-1-transition-passfail-only-score-reporting.

